# Experimental study on deep ultraviolet-C LED disinfection device for often-touch surfaces of advanced medical equipment in hospitals

**DOI:** 10.3389/fpubh.2025.1700856

**Published:** 2026-06-22

**Authors:** Baogen Huang, Feng Wang, Jing Li, Suping Sun, Huiying Lv, Lu Lu, Quan Shi, Chenchen Ding, Yiping Mao

**Affiliations:** 1 Fengxian People's Hospital, Xuzhou, Jiangsu, China; 2 The Affiliated Hospital of Xuzhou Medical University, Xuzhou, China

**Keywords:** Deep LED Ultraviolet-C, disinfection efficacy, hospital infection control, hospital often-touch surfaces, natural microbiota

## Abstract

**Objective:**

To evaluate the disinfection efficacy of a third-generation gallium nitride (GaN)-based deep ultraviolet (UVC) LED disinfection cabinet on often-touch surfaces of small advanced electronic devices in hospital clinical environments, and to provide evidence for its application in hospital infection control.

**Methods:**

A total of 329 device surfaces from 10 randomly selected departments were sampled: 66 computer mice, 151 mobile phones, 53 Personal Digital Assistants (PDAs), 29 electronic glucometers, and 30 electronic sphygmomanometers. Pre-disinfection bacterial contamination levels were assessed. The UVC-LED cabinet was used for disinfection (10-min cycle, 24 mJ/cm^2^ dose), and a natural bacteria eradication test was conducted.

**Results:**

A total of 658 sampling points (329 pre- and post-disinfection) were monitored. Before disinfection, 57.1% of device surfaces showed microbial contamination, with mice and cleaning staff's mobile phones exhibiting relatively severe bacterial contamination. After disinfection, 97.9% of device surfaces showed bacterial colony counts reduced to 0 CFU, with an average natural bacteria eradication rate of 99.9% (range ≥90%).

**Conclusion:**

High-frequency use of small advanced electronic devices by healthcare workers poses hospital infection risks. Deep LED Ultraviolet-C disinfection effectively reduces microbial load on surfaces of devices incompatible with chemical disinfectants or heat. It is recommended as a routine disinfection measure for hospital infection control. This study demonstrates high efficacy under controlled conditions; however, real-world effectiveness may be influenced by factors such as device positioning and surface shadows.

## 1 Introduction

The integration of advanced medical devices with “Internet + informatization” has profoundly transformed clinical workflows, leading to notable gains in diagnostic and treatment efficiency. A new generation of highly integrated electronic devices—such as electronic health record systems, Personal Digital Assistants (PDAs), smartphones, connected glucometers, electronic sphygmomanometers, and barcode scanners—has become ubiquitous in daily hospital operations. While these tools enhance clinical efficiency, they also introduce underappreciated infection control challenges. A Dutch study revealed that over 96% of hospital staff do not regularly clean or disinfect these devices, and 9–27% of them were found to harbor pathogenic microorganisms (1). Research by Dr. Katrina Browne's team demonstrated that focused cleaning and disinfection of shared medical equipment can reduce the incidence of hospital-associated infections by approximately 35% (from 14.9% to 9.8%) (2).

In practice, the surfaces of these advanced devices are frequently touched during clinical use, potentially serving as significant reservoirs for pathogen transmission and elevating the risk of cross-infection (1, 3, 4). Small high-tech electronic devices, being valuable and reusable, require reliable surface disinfection. Clinically, chemical disinfectant wipes are commonly employed but present limitations, including incomplete microbial eradication, potential damage to electronic components, and impractical contact times (5–8). Traditional mercury ultraviolet lamps offer broad-spectrum microbial inactivation but suffer from drawbacks such as mercury content, fragility, large size, and potential ozone generation (5).

Despite the promise of UV disinfection, a significant gap exists in the clinical validation of next-generation UVC-LED technology for disinfecting complex, often-touch electronic surfaces in real-world hospital settings. Current literature lacks robust field studies that connect environmental contamination patterns, device usage behaviors, and the practical efficacy of UVC-LEDs in eliminating naturally occurring microbiota. This study is grounded in the conceptual framework that hospital-acquired infection risk is mediated through contaminated environmental surfaces, and that effective interruption of this transmission pathway requires disinfection modalities that are both microbiologically effective and operationally feasible for delicate electronics.

Over the past decade, LED ultraviolet disinfection technology has emerged in the small-scale UV disinfection market. Building on traditional UV advantages, LED UV offers enhanced clinical prospects due to its electrical characteristics, compact size, versatile configurations, extended lifespan, and mercury-free operation. Notably, LED UV lamps differ from conventional UV lamps in illumination principles and disinfection efficacy, though current research on their clinical application for device surface disinfection remains limited (5, 6, 9). This study aims to fill this gap by investigating bacterial contamination on device surfaces and conducting field tests on a third-generation GaN-based deep UVC-LED disinfection system to analyze its effectiveness, safety, and practicality, thereby providing evidence for reducing hospital-acquired infection risks.

## 2 Materials and methods

### 2.1 Study design

A prospective, controlled before-and-after study design was employed to evaluate the disinfection efficacy of the UVC-LED cabinet on naturally contaminated device surfaces under field conditions in a tertiary hospital.

### 2.2 Experimental materials

**Figure 1:**
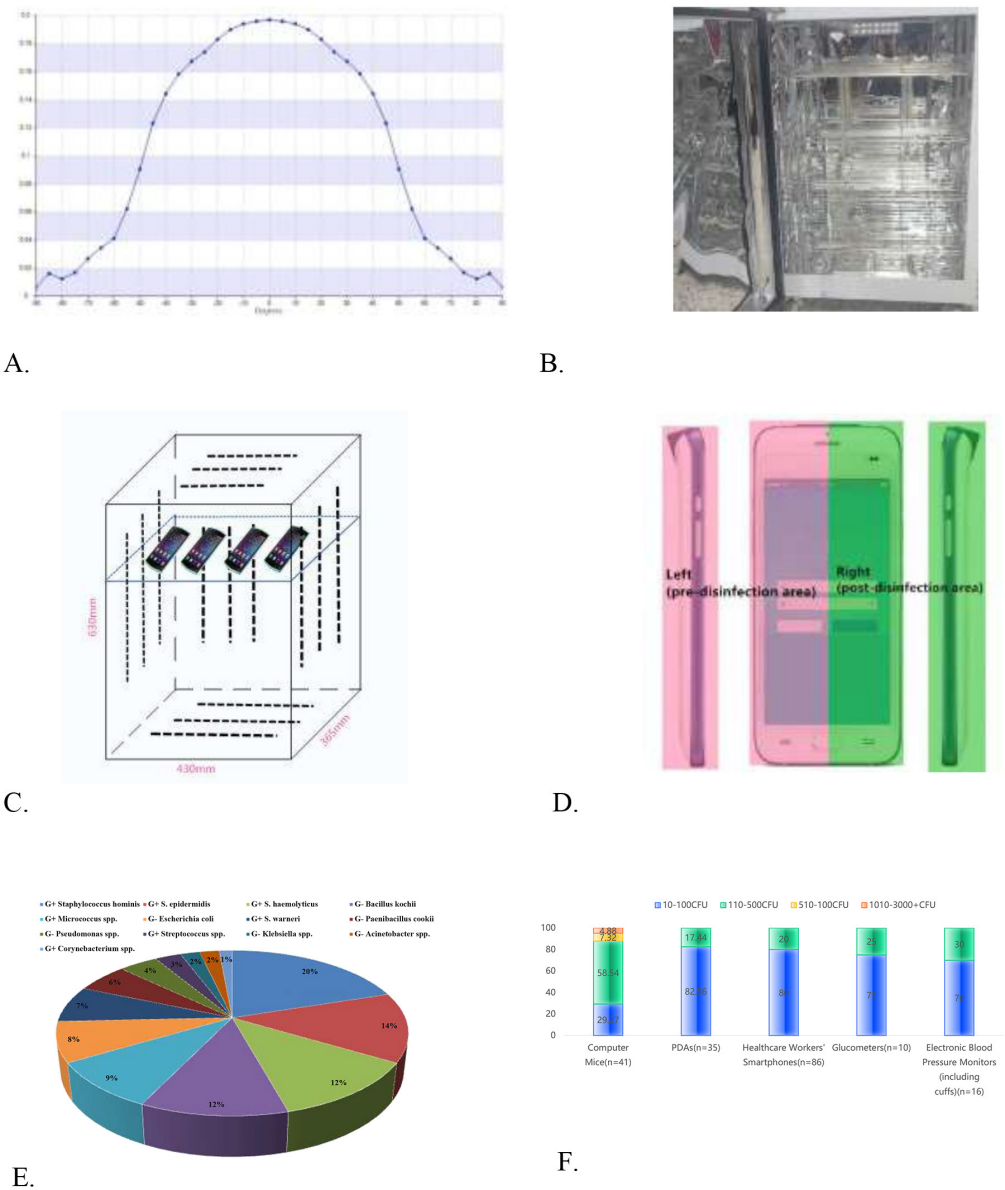
**(A)** LED ultraviolet light distribution curve. **(B)** LED ultraviolet disinfection cabinet device. **(C)** Disinfection point simulation image. **(D)** Sampling area example before and after disinfection. **(E)** Microbial culture and identification of equipment surfaces revealed the distribution of 13 bacterial species (*n* = 140). **(F)** Sampling results of surface pollution of different equipment before disinfection (%).

LED ultraviolet disinfection cabinet, parameters: internal dimensions (length 430 mm × width 365 mm × height 630 mm), LED ultraviolet lamp bead model LY-DUV-TB (size: 3.5 × 3.5 mm), continuous working lifespan: 10,000+ hours, primary wavelength 270~280 nm, intensity 40 μW/cm^2^ (monitored every 6 months). The light distribution and disinfection point layout are illustrated in Figures 1A–C.

Sample collection, culture, and identification: 0.9% sterile saline sampling solution, Sterile cotton swabs, Tryptic Soy Agar (TSA) medium, Constant-temperature incubator, Automated microbial mass spectrometer (Model Autof ms1000), etc.

### 2.3 Study subjects and sampling strategy

Inclusion criteria: Inpatient departments with ≥20 beds in use or high-priority departments for infection control. Using stratified random sampling, 10 departments were selected: three internal medicine departments (Rehabilitation Medicine, Radiation Oncology, Gastroenterology); four surgical departments (Neurosurgery, Orthopedics, General Surgery, Thoracic Surgery); three key infection control departments ( Intensive Care Unit (ICU), Hemodialysis Center, Catheterization Laboratory).

Based on the pre-disinfection surface sampling assessment records (Appendix 1), a purposive sampling strategy was used to select representative, frequently-touched device surfaces from each department, including computer mice, PDAs, glucometers, smartphones, and electronic blood pressure monitors. This approach ensured the inclusion of high-risk surfaces relevant to clinical practice, though it may limit generalizability.

Sampling was performed on the right front, side, and back surfaces of devices before disinfection (after peak clinical activity) and on the left front, side, and back surfaces after disinfection (see Figure 1D for sampling zones). This method was employed to avoid potential cross-contamination between pre- and post-disinfection samples; however, it assumes uniform contamination distribution, which may introduce measurement bias if surfaces are not homogeneously contaminated.

A total of 658 device surfaces (329 pre-and post-disinfection) were sampled: 66 computer mice, 151 smartphones, 53 PDAs, 29 glucometers, 30 blood pressure monitors. The sample size was determined based on departmental availability and the goal of obtaining a representative profile of contamination across major device types and clinical areas, rather than a formal power calculation.

### 2.4 Experimental methods

Disinfection process evaluation: The cabinet's disinfection cycle was standardized at 10-min per device (UV dose 24 mJ/cm^2^), on-site observed and record the process (Appendix 2).

Disinfection efficacy evaluation: Appendix 3 (UV Disinfection Efficacy Evaluation and Testing Record Form) was filled out. Samples were processed within 2 h: The sampling solution was vortexed, and 1.0 mL was inoculated onto TSA plates using the pour plate method (2 plates/sample).The pour plate method was selected for its ability to capture both surface-adherent and embedded microorganisms, and its alignment with standard microbiological protocols for disinfection efficacy testing in hospital settings (10); negative control were included. Plates were incubated at 35~37°C for 48 hours, bacterial identification was performed using an automated microbial mass spectrometer.

Disinfection efficacy formula: $Kill\;rate\;(\text{\%})=\frac{A-B}{A}\times 100$, where A **=** Pre-disinfection colony count (CFU/sample), B **=** Post-disinfection colony count (CFU/sample). Criteria for qualified disinfection: Natural bacteria kill rate ≥90% (log reduction ≥1), with ≥90% of samples meeting this threshold; negative controls show no growth (10–12).

### 2.5 Statistical analysis

Data were organized in Excel2017 and analyzed using SPSS 23.0. Due to the highly skewed distribution of bacterial colony counts, data are presented as median and interquartile range (IQR) in addition to mean ± standard ($\overset{\sim }{x}$ ± s)deviation where appropriate. Non-parametric tests (Mann–Whitney *U*, Kruskal–Wallis) were used for comparisons of bacterial loads. Categorical data were described as counts and percentages (%). A *P*-value < 0.05 was considered statistically significant.

### 2.6 Ethical considerations

This study was approved by the Institutional Review Board (IRB) of [The Medical Ethics Committee of The Affiliated Hospital of Xuzhou Medical University] Approval No: [XYFY2025 - kL106 - 01]. Verbal informed consent was obtained from all healthcare workers whose personal devices (e.g., mobile phones) were sampled, with assurance of data anonymity and that the devices would not be harmed by the sampling or disinfection process.

## 3 Results

### 3.1 Sampling and culture outcomes

Three hundred and twenty-nine pre- and 329 post-disinfection samples were collected, Four negative control cultures showed no growth. One hundred and eighty-eight pre-disinfection samples (57.1%) showed bacterial growth (Table 1). Post-disinfection colony counts were significantly reduced. One hundred and forty-one pre-disinfection samples with no growth were excluded from efficacy rate calculations. Positive samples were rounded to integers for analysis.

**Table 1:** Sampling results and positive culture rates (%) by device type.

Sampling time	Mouse	Cellphone	PDA	BP monitor	Glucometer	Total
Pre-disinfection	62.1% (41/66)	57.0% (86/151)	66.0% (35/53)	53.3% (16/30)	34.5% (10/29)	57.1% (188/329)
Post-disinfection	7.3% (3/41)	0% (0/86)	0% (0/35)	6.25% (1/16)	0% (0/10)	2.1% (4/188)

Negative controls showed no growth. $\;Positive\;rate\;(\text{\%})=\frac{\;number\;of\;culture\;positive\;samples\;}{\;total\;samples\;}\times 100$.

### 3.2 Microbial identification

Among 188 positive cultures, 13 species (140 strains) were identified. Dominant pathogens *Staphylococci* (52.9%): *Staphylococcus hominis, S. epidermidis, S. haemolyticus, S. warneriBacillus* spp.: *Bacillus kochii, Paenibacillus cookii*. Other isolates: *Micrococcus* spp., *Escherichia coli, Pseudomonas* spp., *Klebsiella* spp., *Streptococcus* spp., *Corynebacterium* spp., *Acinetobacter* spp. (Table 2 and Figure 1E).

**Table 2:** Bacterial distribution on device surfaces.

Microorganism	Strains (*n* =1 40)	Proportion (%)
G+ *Staphylococcus hominis*	28	20.00
G+ *S. epidermidis*	19	13.57
G+ *S. haemolyticus*	17	12.14
G- *Bacillus kochii*	16	11.43
G+ *Micrococcus* spp.	13	9.29
G- *Escherichia coli*	11	7.86
G+ *S. warneri*	10	7.14
G- *Paenibacillus cookie*	8	5.71
G- *Pseudomonas* spp.	6	4.29
G+ *Streptococcus* spp.	4	2.86
G- *Klebsiella* spp.	3	2.14
G- *Acinetobacter* spp.	3	2.14
G+ *Corynebacterium* spp.	2	1.43

Overgrown plates due to undiluted samples hindered identification of some species. The mass spectrometer primarily identifies common clinical pathogens and may fail to classify rare isolates.

### 3.3 Pre-disinfection bacterial load and post-disinfection efficacy

Computer mice showed the highest pre-disinfection colony counts (Median: 150 CFU/sample, IQR: 48–380; Mean ± SD: 338 ± 535 CFU/sample), with 12% of samples exceeding 500 CFU and 4.88% surpassing 1,000 CFU. The high standard deviation reflects the extreme skewness of the data, with a few heavily contaminated outliers. PDA devices, healthcare workers' smartphones, and glucometers showed no detectable bacterial growth post-disinfection (colony count = 0 CFU). Computer mice and electronic blood pressure monitors retained minimal residual bacteria post-disinfection (Median: 0 CFU/sample; Mean < 5 CFU/sample) (Table 3 and Figure 1F).

**Table 3:** Comparison of colony counts and natural bacteria elimination efficacy by device type ( ± s).

Device type	Sample size	Pre-disinfection colony count (CFU/sample)	Post-disinfection colony count (CFU/sample)	Bacterial elimination rate (%)
Computer mice	41	150 (48–380); 338 ± 535	0 (0–0); 1 ± 4	99.7 ± 1.2
PDAs	35	55 (30–95); 69 ± 50	0 (0–0); 0	100
Healthcare workers' smartphones	86	45 (20–95); 71 ± 84	0 (0–0); 0	100
Glucometers	10	55 (18–135); 103 ± 135	0 (0–0); 0	100
Electronic BP monitors	16	65 (35–105); 77 ± 57	0 (0–0); 1 ± 3	99.6 ± 1.5

Statistical comparisons (Kruskal–Wallis test) confirmed significant reductions in bacterial load for all device types (*P* < 0.001).

No statistically significant differences were found in bacterial colony counts on mouse surfaces between doctors' offices and nurses' stations across departments (Table 4 toward).

**Table 4:** Bacterial colony contamination on mouse surfaces among staff in different departments and positions ($\overset{-}{x}$ ± s).

Department	Doctor sample size (*n*1 = 22)	Doctors' office	Nurse sample size (*n*2 = 19)	Nurses' station	*t*-value	*P*-value
Internal medicine	8	619 ± 998	7	223 ± 164	1.11	0.30
Rehabilitation medicine	–	350	0	–	–	–
Radiation oncology	3	63 ± 42	3	220 ± 210	1.27	0.30
Gastroenterology	4	1,103 ± 1,295	4	225 ± 156	1.35	0.27
Surgery	11	373 ± 558	10	256 ± 160	0.67	0.51
Neurosurgery	3	827 ± 1,020	4	305 ± 184	0.88	0.46
Orthopedics	2	335 ± 163	1	100	–	–
General surgery	3	93 ± 78	3	160 ± 60	1.18	0.30
Thoracic surgery	3	223 ± 183	2	380 ± 170	0.98	0.42
Key hospital infection departments	3	143 ± 67	2	140 ± 57	0.05	0.96
ICU	1	160	0	–	–	–
Hemodialysis center	2	135 ± 92	0	–	–	–
Catheterization room	0	–	2	140 ± 57	–	–

However, a non-significant trend higher contamination was observed in some doctors' offices (e.g., Gastroenterology), potentially reflecting differences in workflow and cleaning frequency.

Significant differences in pre-disinfection bacterial colony counts on mobile phone surfaces were observed among staff groups (Kruskal–Wallis, *P* < 0.001), with support and cleaning staff showing significantly higher counts than doctors, nurses, and administrative staff (Table 5).

**Table 5:** Bacterial colony contamination on mobile phone surfaces among staff of different positions ($\overset{-}{x}$ ± s).

Position	Sample size (*n*)	Pre-disinfection colony count	Post-disinfection colony count	*F*-value	*P*-value
Doctors	25	51 ± 47	0	15.2	< 0.001
Nurses	25	51 ± 40	0		
Administrative staff	16	26 ± 20	0		
Support/cleaning staff	20	156 ± 126	0		

## 4 Discussion

### 4.1 Key findings and interpretation

This study confirmed significant microbial contamination (57.1% positivity) on often-touch electronic device surfaces in a hospital setting, aligning with international reports (4, 7). The predominance of *Staphylococcus* spp. and the presence of opportunistic pathogens like *E. coli, Klebsiella* spp., *Pseudomonas* spp., and *Acinetobacter* spp. underscore the potential role of these surfaces as reservoirs for nosocomial pathogen transmission (2, 13). The higher contamination on computer mice and support staff phones likely reflects differences in usage patterns, frequency of disinfection, and hand hygiene compliance, suggesting targeted interventions are needed for these high-touch fomites.

### 4.2 Efficacy of UVC-LED disinfection and practical implications

The UVC-LED disinfection cabinet demonstrated high efficacy, achieving a 100% eradication rate (0 CFU post-disinfection) on PDAs, healthcare worker smartphones, and glucometers, and an overall average eradication rate of 99.9%. These results are consistent with a growing body of evidence supporting the efficacy of UVC-LED technology against a broad spectrum of microorganisms (8, 9, 14–21). The minimal residual contamination (< 5 CFU/sample) found on computer mice and blood pressure monitor cuffs is likely attributable to shadowing effects in complex geometries (e.g., mouse wheels, folded cuff surfaces) (1, 9, 17, 22), highlighting a key operational consideration.

Compared to chemical disinfectants, which can be corrosive, require long contact times, and leave potentially harmful residues (5–8), and traditional mercury UV lamps, which contain hazardous materials and have operational limitations (5), the UVC-LED cabinet offers a dry, chemical-free, and user-friendly alternative. Its 10-min cycle time, instant start-up, and compatibility with delicate electronics present a significant practical advantage for integration into busy clinical workflows without damaging equipment (23–25).

### 4.3 Strengths, limitations, and research context

This study, as a component of the research project “Research and Development of Deep Ultraviolet LED Hospital Environment Disinfection Technology Based on Third-Generation Semiconductor Gallium Nitride and Multicenter Study on Disinfection Efficacy,” has the following strengths: First, advanced technology: it pioneers the application of GaN-based deep UVC-LED technology for disinfecting hospital equipment surfaces, validating its feasibility. Second, clinically relevant design: it assesses devices across multiple departments via field study and identifies natural microbiota profiles. Third, significant efficacy: an average eradication rate of 99.9% establishes a foundation for subsequent research. The limitations of this study simultaneously outline directions for further development within the project: First, the single-center design highlights the necessity of the subsequent “multicenter study” to verify generalizability. Second, the lack of long-term compatibility assessment is a crucial step for the technology's transition to mature application. Third, the direct impact on hospital infection rates remains unverified, requiring more rigorous studies to build a complete evidence chain. Thus, this study accomplishes a key step from technology development to single-center efficacy verification. Its limitations precisely provide a clear starting point and rationale for the project's subsequent phases: multicenter validation, long-term safety assessment, and benefit analysis.

## 5 Conclusion

The widespread integration of high-tech medical devices, while essential for modern healthcare, introduces significant nosocomial infection risks through contamination of often-touch surfaces. this study provides strong evidence supporting the effectiveness of UVC-LED technology in reducing microbial bioburden on these critical surfaces. Specifically, it confirms that a deep UVC-LED disinfection cabinet is a highly effective, user-friendly, and sustainable solution for mitigating microbial contamination across a variety of electronic device surfaces in hospitals. Its implementation can significantly enhance infection control protocols, especially where chemical disinfection or traditional UV methods are inadequate. Therefore, the adoption of this technology in clinical settings is recommended.

## Data Availability

The original contributions presented in the study are included in the article/Supplementary material, further inquiries can be directed to the corresponding author.
