# Supplementary material for: Experimental study on deep ultraviolet-C LED disinfection device for often-touch surfaces of advanced medical equipment in hospitals
Source: Front Public Health. 2026 Jun 22;13:1700856. doi: 10.3389/fpubh.2025.1700856 (PMC13335072; doi:10.3389/fpubh.2025.1700856)
Supplement: Supplementary file 1 [file Data_Sheet_1.docx]

Appendix Table 1

Pre-Disinfection Surface Sampling Assessment Form for Hospital Environments

| No. | Object | Material | Sampling Timing | Quantity |
| --- | --- | --- | --- | --- |
| 1 | Mobile phones (doctors, nurses, support staff) |  |  |  |
| 2 | PDAs (nurses) |  |  |  |
| 3 | Computer mice (doctors, nurses) |  |  |  |
| 4 | Blood pressure monitor and cuff (doctors, nurses, patients) |  |  |  |
| 5 | Glucometer |  |  |  |
| … | …… |  |  |  |
| Material types: Glass, plastic, rubber, stainless steel, paper, fabric, wood, leather, etc.  Note: Microbial load and survival time may vary by material type, potentially affecting disinfection efficacy. | | | | |

Appendix Table 2

UV Disinfection Work Log

| No. | | | | | |
| --- | --- | --- | --- | --- | --- |
| Disinfection Device |  | Intensity Monitoring Method | |  | |
| Disinfection Location |  | Disinfection Date | |  | |
| Disinfection Object | Area/Quantity | Temperature & Humidity | Disinfection Method | | Exposure Time |
|  |  |  |  | |  |
|  |  |  |  | |  |
|  |  |  |  | |  |
| …… |  |  |  | |  |
| Additional Notes: | | | | | |
| - Performed by: ___________________  - Evaluated by: ___________________  - Evaluation Result: ☐ Qualified / ☐ Unqualified  - Evaluation Date: ________ (Month/Day/Year) | | | | | |

Appendix Table 3

UV Disinfection Efficacy Evaluation: Sampling and Test Results

| No. | | | | | | |
| --- | --- | --- | --- | --- | --- | --- |
| Location/Object |  | | | | | |
| Disinfection Date |  | Exposure Time |  | Sampler |  | |
| Test Date |  | Report Date |  | Sampling Time |  | |
| Sample Name | Pre-Disinfection Sample | | | Post-Disinfection Sample | | |
|  | Sample ID | Result | Judgment | Sample ID | Result | Judgment |
|  |  |  |  |  |  |  |
|  |  |  |  |  |  |  |
|  |  |  |  |  |  |  |
| …… |  |  |  |  |  |  |
| Additional Notes: | | | | | | |
| Additional Notes:  - Tested by: ___________________  - Reviewed by: ___________________  - Received by: ___________________  - Date: ________ (Month/Day/Year) | | | | | | |
